# Case Report: unexpected cause of cyanosis in an infant after acute exposure to high altitude—severe tricuspid regurgitation secondary to tricuspid valve prolapse

**DOI:** 10.3389/fcvm.2024.1335218

**Published:** 2024-06-12

**Authors:** Yaru Cui, Lixia Yang, Ping Wu, Shuran Shao, Shuhua Luo, Kaiyu Zhou, Xiaoliang Liu, Chuan Wang, Hongyu Duan

**Affiliations:** ^1^ Department of Pediatrics, West China Second University Hospital, Sichuan University, Chengdu, Sichuan, China; ^2^ The Cardiac Development and Early Intervention Unit, West China Institute of Women and Children’s Health, West China Second University Hospital, Sichuan University, Chengdu, Sichuan, China; ^3^ West China Medical School of Sichuan University, Chengdu, Sichuan, China; ^4^ Key Laboratory of Birth Defects and Related Diseases of Women and Children of MOE, Department of Pediatrics, West China Second University Hospital, Sichuan University, Chengdu, Sichuan, China; ^5^ Key Laboratory of Development and Diseases of Women and Children of Sichuan Province, West China Second University Hospital, Sichuan University, Chengdu, Sichuan, China

**Keywords:** tricuspid regurgitation, tricuspid valve prolapse, rupture, chordae tendinea, infant, surgery, high altitude

## Abstract

**Background:**

Severe tricuspid regurgitation (TR) causing cyanosis with patent foramen ovale (PFO) and right-to-left atrial shunting requires a precise diagnosis for optimal therapy. Tricuspid valve prolapse (TVP) can lead to TR and is sometimes overlooked, especially in complex cases with factors like pulmonary hypertension (PH). We present an infant with cyanosis and profound TR after high-altitude exposure, initially misattributed to PH but found to be primarily due to spontaneous chordae tendineae rupture and TVP. This case underscores the challenges in diagnosing TR-induced cyanosis.

**Case presentation:**

The 3-month-old infant rapidly developed cyanosis, hypoxemia, right atrial enlargement, severe tricuspid regurgitation (TR), and patent foramen ovale (PFO) shunting after high-altitude exposure. Although echocardiography revealed tricuspid valve prolapse (TVP), initial consideration linked TR and right-to-left shunting to pulmonary hypertension (PH) due to the temporal correlation with rapid altitude exposure. Despite hemodynamic stability and the absence of respiratory distress after respiratory support and combined PH medication therapy, the persistent hypoxemia did not reverse as expected. This treatment outcome and repeated echocardiograms reminded us that TR was primarily caused by TVP rather than PH alone. Intraoperative exploration confirmed that TVP was caused by a rupture of TV chordae tendineae and anterior papillary muscle head, and the chordae tendineae/papillary muscle connection was reconstructed. After surgery, this patient was noncyanotic with an excellent long-term prognosis, a trivial TR with normal TV function being observed echocardiographically.

**Conclusions:**

TR-induced cyanosis can be not only a consequence of PH and right-sided heart dilation but also a primary condition. Repetitive reassessment should be undertaken with caution, particularly when patients are not improving on therapy in the setting of conditions known to predisposition to secondary TR. Since TVP caused by rupture of the chordae or papillary muscles is rare but fatal in children, early diagnosis is clinically substantial to proper management and satisfactory long-term outcomes.

## Introduction

Central cyanosis indicates hypoxemia and may signal life-threatening conditions requiring urgent treatment due to serious hemodynamic abnormalities. Identifying the underlying causes of cyanosis promptly is crucial for appropriate management. In children, the causes can generally be classified as pulmonary, cardiac, hematological, or metabolic (1). In terms of cardiac causes, advanced tricuspid regurgitation (TR) can lead to cyanosis, especially with patent foramen ovale (PFO) or atrial septal defects causing right-to-left atrial shunting. TR is typically secondary to significant right ventricular (RV) dilation and dysfunction in conditions like right ventricular outflow tract obstruction and pulmonary hypertension (PH), resulting in tricuspid annulus (TA) dilation, leaflet tethering, and malcoaptation (2–4).

On the other hand, primary TR, characterized by isolated damage to the tricuspid valve (TV) apparatus (including tricuspid leaflets, annulus, chordae, or papillary muscles), accounts for only 8%–10% of TR cases and is often overlooked (5). This type of TR is commonly associated with endocarditis, Ebstein anomaly, tumors, iatrogenic injury, trauma, and autoimmune diseases (6–10). However, chordal and papillary muscle rupture leading to tricuspid valve prolapse (TVP) is an extremely rare but emergent cause of TR (11). Moreover, this condition is more likely to be missed, particularly in pediatric patients with complicating factors that may steer the diagnosis toward secondary TR such as PH (12, 13), potentially leading to a poor prognosis. Here, we present a unique case of an infant with refractory central cyanosis complicated by profound TR, initially attributed solely to PH but predominantly caused by spontaneous rupture of chordae tendineae and TVP.

## Case descriptions

The 3-month-old male infant experienced continuous cyanosis, fatigue, and sweating for 12 hours after rapidly ascending from a plain region to an altitude of 3,200 meters within 3 h (the altitude ascent speed is nearly 1,000 meters per hour). Despite immediate descent to a lower altitude, and treatment with oxygen, digitalis, and diuretics, his condition worsened, necessitating transfer to our hospital's cardiac intensive care unit (at an altitude of 500 meters) by ambulance. On arrival, a detailed clinical history ruled out fever, perinatal asphyxia, maternal complications during pregnancy, and a family history of congenital heart disease or other hereditary diseases. A history of chest trauma was also denied by his parents.

The patient showed irritability, cold extremities, tachycardia (179 beats/min), tachypnea (52 breaths/min), hypotension (65/40 mmHg), and cyanosis, despite receiving oxygen at 5 L/min via a face mask, with a pulse oxygen saturation of 89%. A normal pulmonic second heart sound and a soft (Ⅰ/Ⅵ) systolic murmur were noted in the left second and fourth intercostal spaces, respectively. Pulmonary rales were evident, but there were no signs of abdominal distention, hepatomegaly, facial swelling, or leg edema. No unusual facial or external features were observed. Laboratory tests revealed elevated brain natriuretic peptide levels of 5,175.1 pg/ml (reference range, 0–100 pg/ml) and myocardial troponin I levels of 0.11 µg/L (reference range, 0–0.06 µg/L). Blood gas analysis showed a pH of 7.458, arterial oxygen saturation (SaO2) of 83.5%, pO2 of 44.5 mmHg, pCO2 of 28.8 mmHg, and lactate of 3.06 mmol/L. Complete blood cell count, erythrocyte sedimentation rate, C-reactive protein level, urinalysis, liver and renal function, electrolytes, and two pairs of blood cultures were within normal limits. Both chest x-ray and enhanced chest CT indicated right atrial enlargement with dark lung fields, without evidence of intravascular thrombosis or abnormalities in the pulmonary arteries or veins. An electrocardiogram showed sinus tachycardia along with right atrial enlargement. Despite normal signs of segmental heart anatomy, annular attachments, coronary sinus, venous connection, and systolic function, the initial echocardiogram revealed right atrial (22 mm × 26 mm) and ventricular (13 mm) enlargement, along with systolic prolapse of the anterior and posterior leaflet across the tricuspid plane. Color flow mapping showed a profound tricuspid regurgitation (TR) jet-directed towards the patent foramen ovale (PFO) with a velocity of 2.1 m/sec and a peak gradient pressure of 18 mmHg. No pulmonary regurgitation (PR) was detected.

Although TVP was visualized echocardiographically, TR and right-to-left atrial shunting was still expected to be correlated with PH in view of the remarkable concordance between disease onset and acute exposure to high-altitude hypoxia. The patient was diagnosed with high-altitude PH (HAPH) complicated by the pulmonary hypertensive crisis (PHC), immediately receiving treatment with high-flow nasal oxygen (HFNO) and bosentan (4 mg/kg per day), plus continuous infusions of milrinone (0.75 µg/kg/min) and furosemide (0.1 mg/kg/h). Meanwhile, he required norepinephrine (0.3 µg/kg/min) and vasopressin (0.04 U/kg/min) for maintenance of systemic blood pressure. However, the respiratory status of this patient was exacerbated, and intubation and mechanical ventilation were instituted promptly. Other therapies, including complete sedation/analgesia, topical cooling of the head for brain protection, and sodium bicarbonate infusion, were applied. Discouragingly, marked hypoxemia was persistent with SaO2 of 70% and pO2 of 35 mmHg even under the administration of 100% oxygen. Thereafter, repeated chest CT and bronchoscopy were undergone for comprehensive airway evaluation, illustrating that respiratory diseases were not the major causes of hypoxemia. Extensive laboratory tests, including coagulation profile, autoantibodies, HIV testing, genome sequencing, and metabolic disorder screening, further excluded other causes of PH. Then, nitric oxide (20 ppm), tadalafil (1 mg/kg per day), and continuous infusion of treprostinil (starting dose of 1.25 ng/kg/min and titrated to 10 ng/kg/min as tolerated) were administered under close monitoring. His clinical status was greatly improved in the context of systemic hypotension, tachycardia, and disturbed internal milieu; however, intractable hypoxia failed to be reversed as expected, ranging in SaO2 from 74% to 86%. This frustrating consequence ultimately raised our concerns that the major culprit of hypoxemia owing to massive TR seemed to be other potential causes rather than PH. Actually, some important clues undoubtedly confirmed the suspicion of this possibility after a review of the clinical scenario in this case: (1) scant evidence of “right heart failure,” including hepatomegaly, pleural effusions, and ascites, (2) inconsistency in acidosis and cardiopulmonary collapse, (3) unexpectedly prolonged duration of respiratory support along with the discrepancy between improvements in hemodynamic status and persistence of hypoxemia, and (4) massive TR but without correspondingly echocardiographic visualization of high-velocity TR jet, pulmonary artery dilation, prominent RV enlargement, and septal flattening.

**Figure 1:**
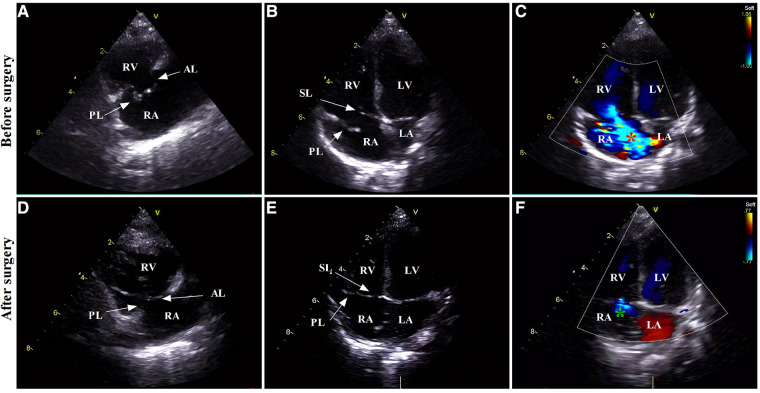
The transthoracic echocardiographic images before and after surgical repair. Preoperative RV inflow (**A**) and apical four-chamber views (**B,C**) showing dilation of RA/RV, systolic prolapse of AL/PL toward the RA (white arrows), and severe TR with right-to-left shunting through PFO (red asterisk). Postoperative echocardiogram (**D–F**) showing normalized RA/RV, complete edge-to-edge leaflet coaptation (white arrows), and trivial TR (green asterisk). RV, right ventricle; RA, right atrium; LA, left atrium; LV, left ventricle; AL, anterior leaflet; PL, posterior leaflet; SL, septal leaflet; TR, tricuspid regurgitation; PFO, patent foramen ovale.

**Figure 2:**
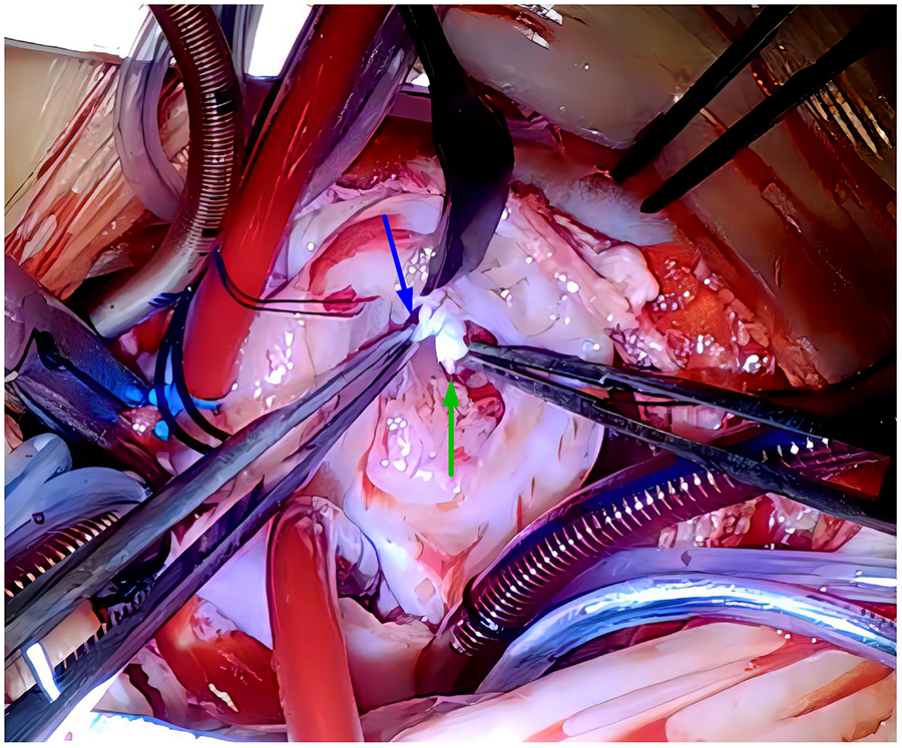
Intraoperative findings showing ruptures of the TV chordae tendineae (blue arrow), and head of anterior papillary muscle (green arrow). No fibromyxomatous changes were observed. TV, tricuspid valve.

Courageously, discontinuation of combination pharmacotherapy for PH and mechanical ventilation had been undergone on hospital day 12. Indeed, this patient was hemodynamically stable without breathlessness and progressive deterioration of cyanosis. A repeated echocardiogram once again showed ongoing and massive TR directed to the interatrial septum secondary to TVP with a peak gradient pressure of 21.9 mmHg, severely dilated RA (24 mm × 29 mm), and right to left shunting through the enlarged PFO (5 mm diameter). Of note, the pulmonary trunk was narrowed (7.7 mm diameter) attributable to compromised antegrade flow from the RV toward the pulmonary artery, mimicking pulmonary atresia (Figures 1A–C). The right ventricular outflow tract acceleration time was also measured this time and the value was 120 ms (reference value ≥105 ms). These echocardiographic characteristics reminded us again that TR in the present case would predominantly result from TVP but not PH alone. This patient was finally referred for surgery to uncover the cardiac lesions on hospital day 17. During surgery, the prolapsed and flailed leaflets of anterior and posterior tricuspid valves were visualized immediately after a right atriotomy, while the septal leaflets and the medial papillary muscle were normal. During further exploration, the primary chordae tendineae was ruptured at the head of the anterior papillary muscle, conferring the absence of subvalvar apparatus supporting the involved leaflets (Figure 2). The pulmonary pressure measured on the operating table was 21/10 mmHg, which was normal and further proved the cyanosis mainly resulted from tricuspid chordae rupture and severe TR. The papillary muscle seemed to have ischemic lesions, and no fibromyxomatous changes being identified. The chordae were reconnected to the anterior papillary muscle head via a 6–0 pledgeted polypropylene suture. The tricuspid valve was further supported by Clover Technique to mitigate the future risk of recurrent regurgitation. The saline test was satisfactory. Subsequently, cardiopulmonary bypass was weaned successfully. After surgery, cyanosis was completely reversed, while mechanical ventilatory and inotropic supports were withdrawn within the first 36 h. An echocardiogram on postoperative day 9 showed a trivial TR with complete edge-to-edge leaflet coaptation, favorable ventricular contractility, and normal size of the proximal main pulmonary artery and cardiac chambers (Figures 1D–F). He was discharged on postoperative day 10. The last visit at 5 months old displayed normal development without any neurological sequela. (The specific echocardiogram data are shown in Table 1).

**Table 1:** The preoperative and postoperative echocardiographic data of the children.

	Before surgery	After surgery
The diameter of RA (mm)	29 (Z-Score 3.21)	20 (Z-Score −0.7)
The diameter of RV (mm)	17	9.5
TR	severe TR	Trivial TR
TR velocity (m/s)	Vmax = 1.6	Vmax = 1.8
TVP	Systolic prolapse of the anterior and posterior leaflet across the tricuspid plane	Tricuspid valvuloplasty was performed
Direction of shunt at the PFO	Right-to-left shunt	The PFO suture was performed
The inner diameter of MPA (mm)	7.7	12

The diameter of RA: the diameter of the long axis in apical four-chamber cardiac view; The diameter of RV: the inner diameter of right ventricular outflow tract in parasternal left ventricular outflow tract view.

TR, tricuspid regurgitation; TVP, tricuspid valve prolapse; PFO, patent foramen ovale; MPA, main pulmonary artery.

## Discussion

An important, although uncommon, clinical consequence of massive TR is the development of hypoxemia via right-to-left shunting. Considering its excess mortality and heterogeneity of etiology, addressing the underlying disease of TR is the cornerstone for defining the most appropriate therapeutic strategy in patients with this condition (14). Herein, we described a rare pediatric case of TR-induced intractable hypoxemia mainly caused by TVP. This report highlighted the clinical challenges and significance in the differential diagnosis for the pathology of TR, which may occur not only as a consequence of PH and right-sided heart dilation but also as a primary condition. More importantly, repetitive reassessment should be undertaken with caution, particularly when patients are not improving on therapy in the setting of conditions known to predisposition to TR.

As for our case, a clear history of acute exposure to high altitude was an important confounding factor that diverted the etiologic diagnosis of TR initially toward HAPH complicated by PHC alone. However, a variety of clinical scenarios as follows were not consistent with the hemodynamic features of this condition, which was ignored at the early stage of the disease course and resulted in the delayed diagnosis. First, as poor tolerance of RV to cope with brisk increases in pressure loading, the right heart failure would be more prominent, heralded by systemic venous congestion, edema, hepatomegaly, ascites, pleural effusions, and pericardial effusions. It was also unexpected in this hemodynamic status that the RV size didn't increase proportionately with that of RA, contributing to the mild enlargement of RV and TA. Of note, considering the ventricular and annular dilations were the primary factors triggering TR in cases of PH, the massive TR didn't seem to be produced by PH alone in terms of the inconsistent correlation between the degree of morphological alterations and severity of TR (15–17). Second, it has been reported that the velocity of the TR jet is not proportional to the severity of PH in cases with severe TR due to altered hemodynamic interaction of right heart structures (18). Thus, considering high altitude exposure before disease onset and subsequent hemodynamic instability in our case, PHC could not be excluded despite the low velocity of the TR jet. However, the absence of leftward displacement/flattening of the interventricular septum left ventricle compression, and pulmonary artery dilation didn't correspond with the echocardiographic features of PHC. Third, paradoxically, the patient had severe hypoxemia but without any radiographic findings of pulmonary edema.

Furthermore, hypoxemia was poorly responsive to prolonged mechanical ventilation and combination pharmacotherapy for PH; on the other hand, TR, as well as hypoxemia, had never been relieved over time as expected, despite dramatic improvement in cardiopulmonary compromise, implying TR severity was not associated with alleviation of PH. A preliminary diagnosis of PH could not satisfactorily explain all the aforementioned phenomena related to massive TR, favoring a high index of suspicion of anatomic substrate for the origin of TR, namely TVP. Virtually, TVP contributes to pure volume overload of the right heart, and therefore, a normal RV exhibits good tolerance to this condition without excessive dilatation/dysfunction and TA dilation (19). Failure of leaflet coaptation due to rupture on the subvalvular apparatus can generate wide open TR during systole, creating rapid equalization of RV and RA pressures and, in turn, low-velocity TR jet (20). Given the restricted motion of prolapsed leaflets, the backflow is preferably directed toward dilated PFO into the systemic circulation. These factors contribute to severe eccentric TR, flow-driven right-to-left shunting, compromised antegrade pulmonary flow, and profound hypoxemia (5, 15, 21). However, it should be noted that coexisting PH that augments the vicious circle of cardiopulmonary collapse may still need to be considered (at least in the early stage of disease onset), according to the clinical features of this case.

With scarce published data in the pediatric population, TVP tends to be a forgotten mechanism of cyanosis in children. To the best of our knowledge, TVP-induced cyanosis has merely been observed in neonatal patients with or without congenital heart anomalies (26 reported cases), whose pulmonary vascular resistances have not yet fallen to adult levels, thus facilitating severe TR and the right to left shunting through PFO (13, 22–25). However, our case emphasized this phenomenon could also be found even in previously healthy infants after environmental exposure predisposing to TVP. Based on the exclusion of primary congenital anomalies of the TV apparatus, TVP was believed to be secondary to the rupture of chordae tendineae in our case. This lesion has been reported to be caused by ischemic etiologies, including hypoxemia, myocardial infarction, connective tissue diseases, thromboembolism, fibromyxomatous changes, and rhesus isoimmunization, as well as non-ischemic etiologies, such as infectious endocarditis, trauma, endomyocardial biopsy (26–31). However, it is noteworthy that pressure overload of the RV can also cause rupture of the chordae or papillary muscles. This phenomenon has been described in cases with premature closure of the ductus arteriosus *in utero*, persistent fetal circulation, PH, and congenital heart defects such as dextro- or levo-transposition of the great arteries, and tetralogy of Fallot (13, 22, 32–34), which account for high pressure in the systolic phase and pronounced mechanical stress on both the valvular and subvalvular apparatus.

Additionally, considering profound coronary blood flow during systole in the RV as opposed to the LV, the systolic overload and resultant elevated ventricular wall tension with decreased myocardial perfusion pressure makes the anterior papillary muscle more susceptible to ischemia due to its considerable demand for oxygen and blood supply from the distal branches of the coronary circulation (19). In the present case, despite the unknown mechanisms, the acute development of PH might offer a possible explanation for the rupture after the exclusion of the heterogeneous spectrum of related diseases. Moreover, hypoxic-ischemic conditions also seemed to participate in the valvular and cardiac dysfunctions. Collectively, the presence of the aforementioned predisposing factors may have ultimately resulted in disastrous consequences.

## Data Availability

The original contributions presented in the study are included in the article/Supplementary Material, further inquiries can be directed to the corresponding authors.
